# Supplementary material for: Quantitative proteomics identifies tumour matrisome signatures in patients with non-small cell lung cancer
Source: Front Oncol. 2023 Jun 16;13:1194515. doi: 10.3389/fonc.2023.1194515 (PMC10313119; doi:10.3389/fonc.2023.1194515)
Supplement: Supplementary file 7 [file DataSheet_7.docx]

**Supplementary Information**

**Quantitative proteomics identifies tumour matrisome signatures in patients with non-small cell lung cancer**

Helen F. Titmarsh, Alex von Kriegsheim, Jimi C. Wills, Richard A. O’Connor, Kevin Dhaliwal, Margaret C. Frame, Samuel B. Pattle, David A. Dorward, Adam Byron, Ahsan R. Akram*

Contents:

Supplementary Figure 1

Supplementary Figure 2

Supplementary Figure 3

Supplementary Figure 4

Supplementary Table 1

Supplementary Table 2

Supplementary Table 3


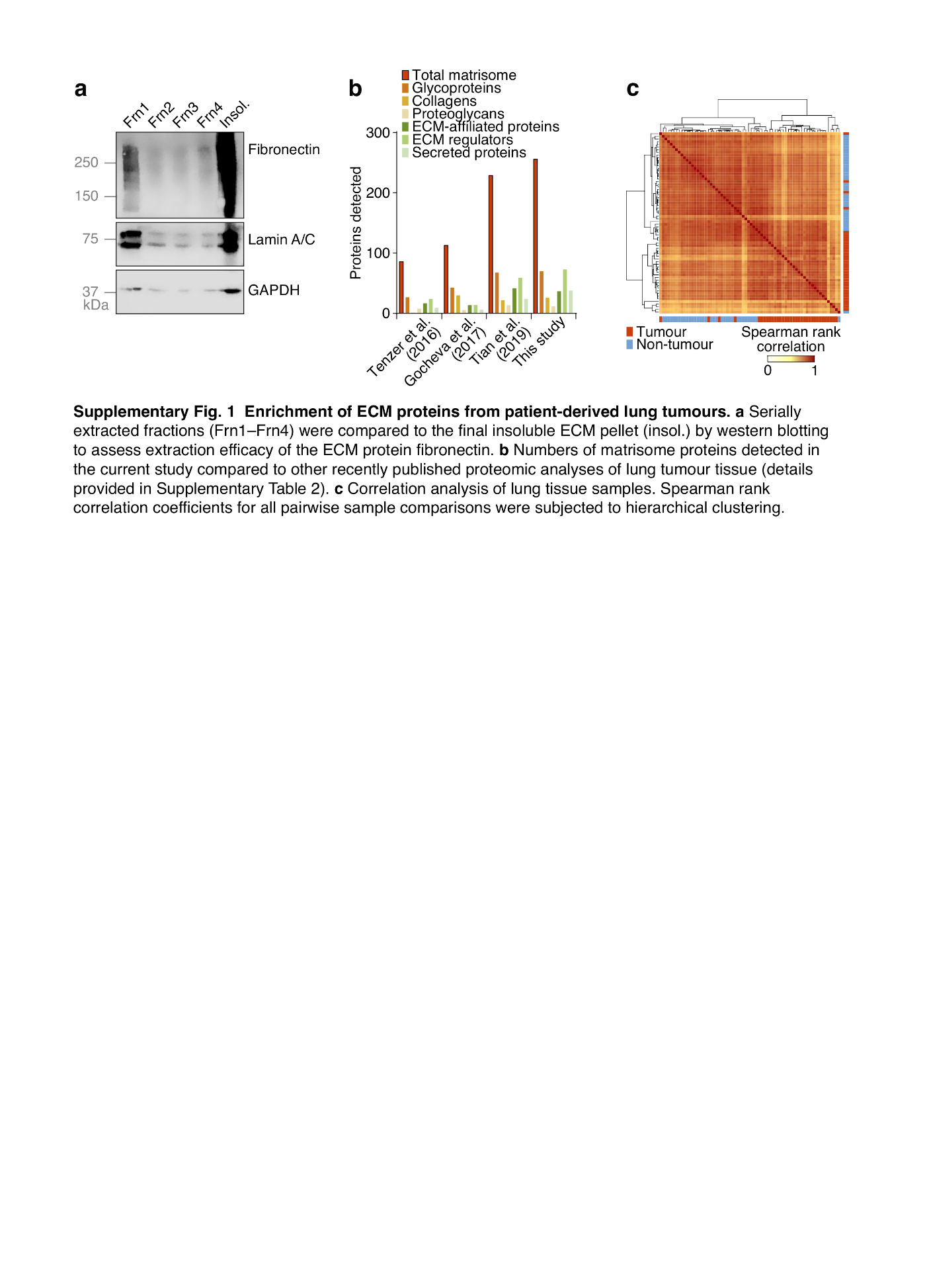


**Supplementary Fig. 1 Enrichment of ECM proteins from patient-derived lung tumours. a** Serially extracted fractions (Frn1–Frn4) were compared to the final insoluble ECM pellet (insol.) by western blotting to assess extraction efficacy of the ECM protein fibronectin. **b** Numbers of matrisome proteins detected in the current study compared to other recently published proteomic analyses of lung tumour tissue (details provided in Supplementary Table 2). **c** Correlation analysis of lung tissue samples. Spearman rank correlation coefficients for all pairwise sample comparisons were subjected to hierarchical clustering.


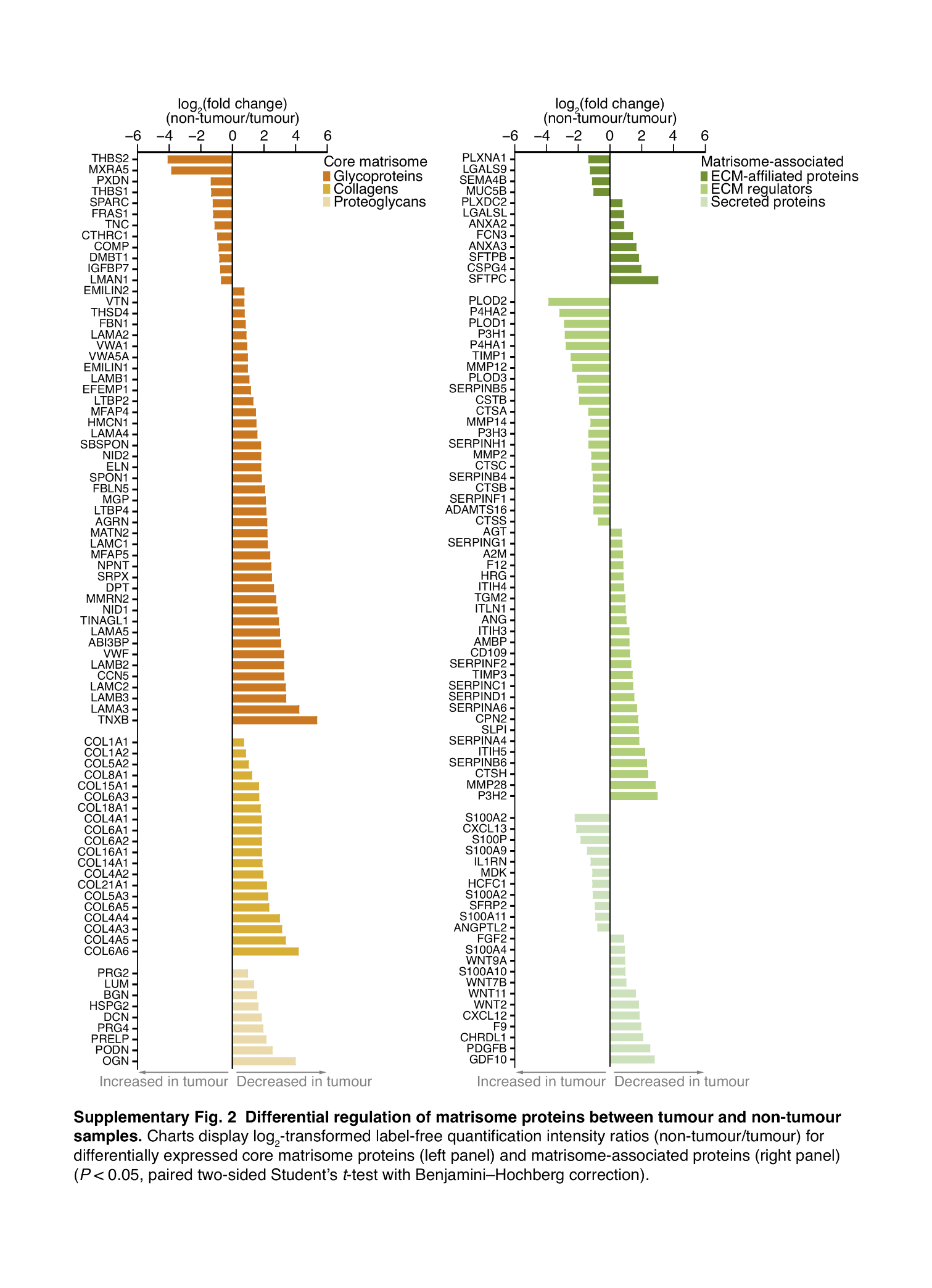


**Supplementary Fig. 2 Differential regulation of matrisome proteins between tumour and non-tumour samples.** Charts display log2-transformed label-free quantification intensity ratios (non-tumour/tumour) for differentially expressed core matrisome proteins (left panel) and matrisome-associated proteins (right panel) (*P* < 0.05, paired two-sided Student’s *t*-test with Benjamini–Hochberg correction).


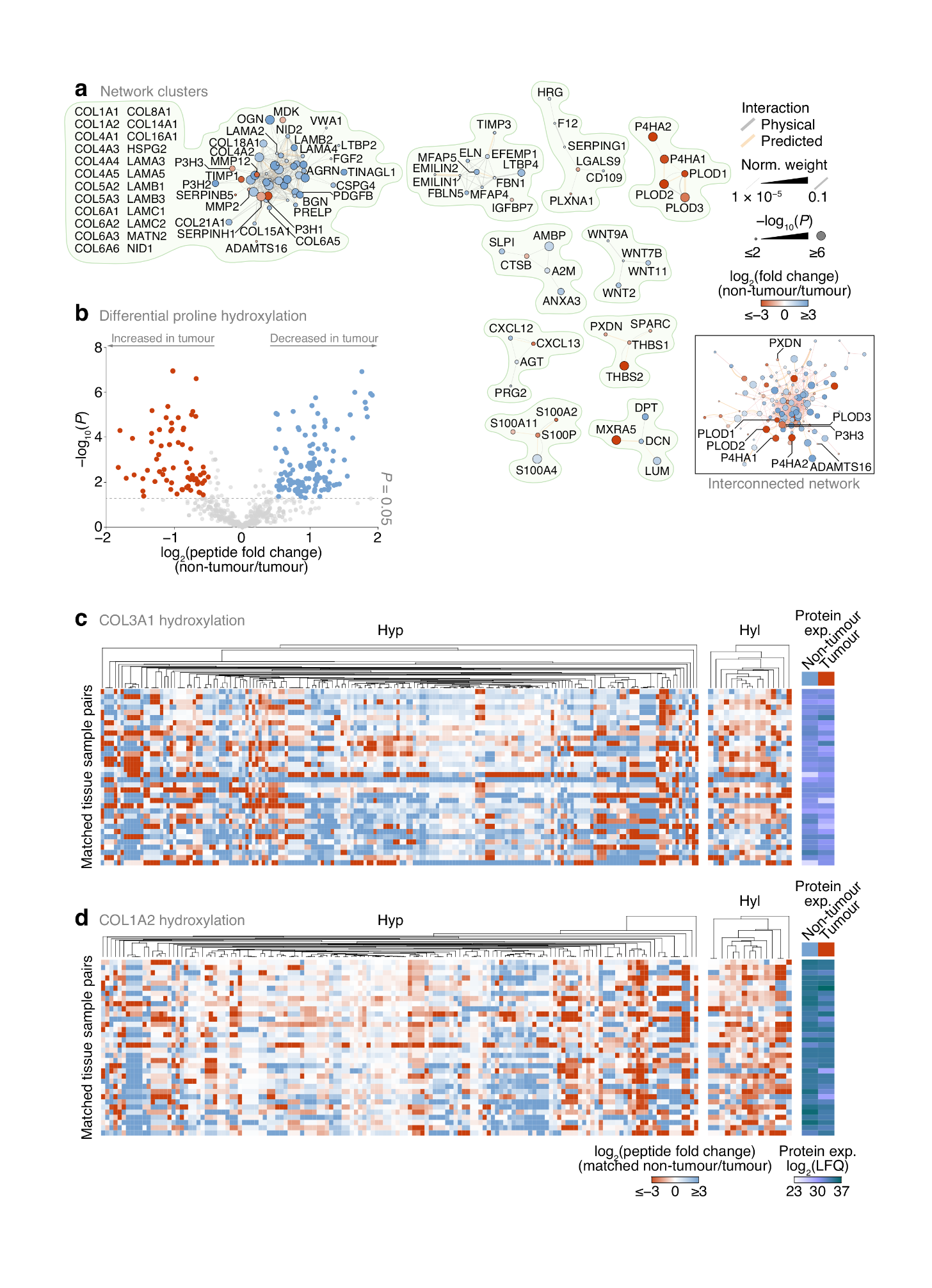


**Supplementary Fig. 3 Regulation of proline and lysine hydroxylation in patient-derived lung tumour ECM. a** Markov clustering of the interaction network of proteins differentially regulated in tumour and nontumour ECM (P < 0.05, paired two-sided Student’s t-test with Benjamini–Hochberg correction). Proteins (nodes) are coloured according to enrichment or depletion in tumour samples and sized according to statistical significance. Protein interactions (edges) were weighted according to evidence of co-functionality. Clusters with at least four proteins are shown. Inset, complete connected network. **b** Volcano plot of peptides containing hydroxylated proline quantified by MS-based proteomics. Differentially regulated peptides are indicated with large coloured circles (red, increased in tumour; blue, decreased in tumour) (P < 0.05, FDR 20%, paired twosided Student’s t-test with Benjamini–Hochberg correction). **c, d** Regulation of proline and lysine hydroxylation in type III collagen α1 chain (COL3A1) (**c**) and type I collagen α2 chain (COL1A2) (**d**) across 34 matched nontumour– tumour paired tissue samples. Total protein expression determined by label-free quantification (LFQ) shown for corresponding samples.


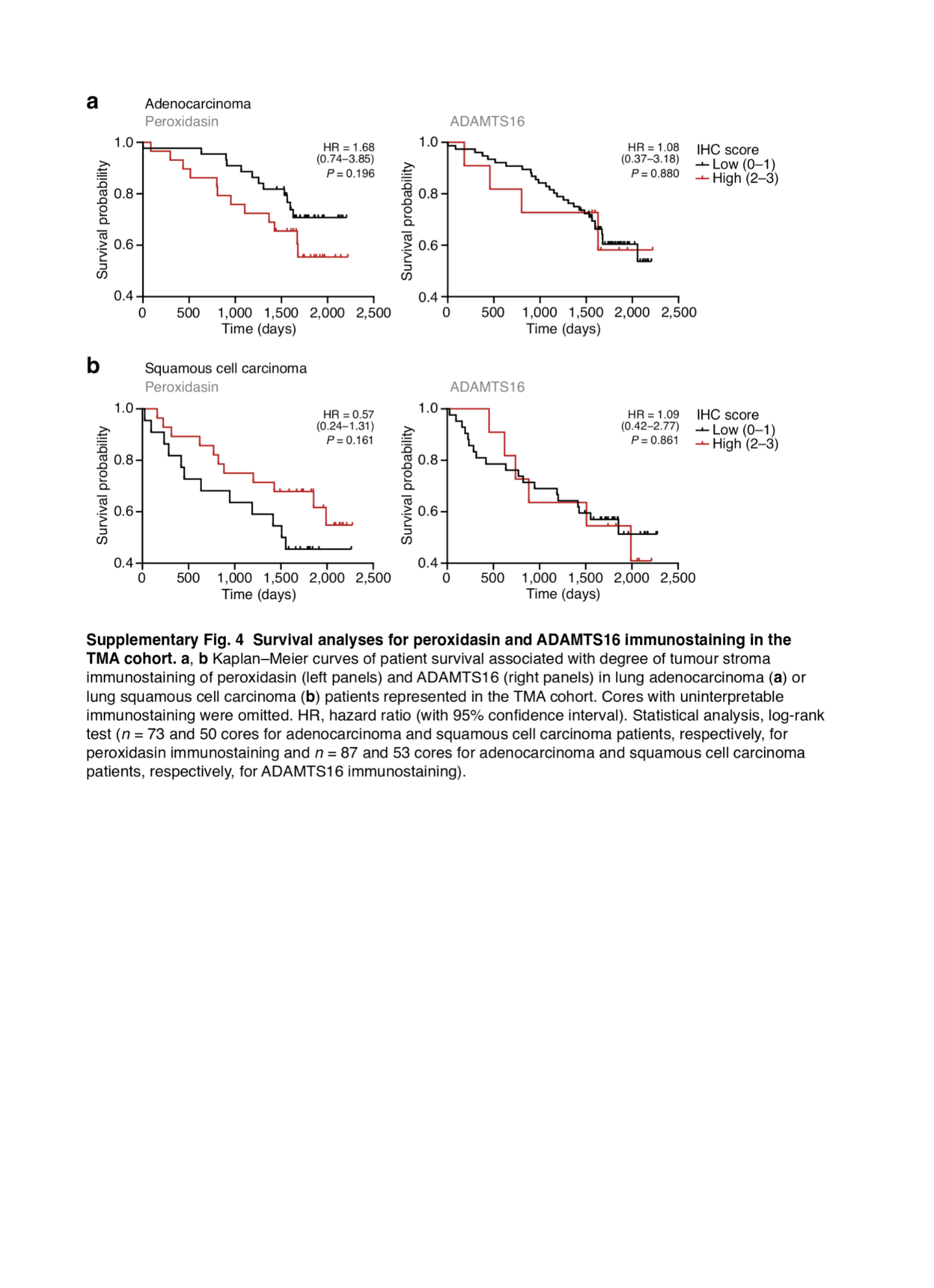


**Supplementary Fig. 4 Survival analyses for peroxidasin and ADAMTS16 immunostaining in the TMA cohort. a**, **b** Kaplan–Meier curves of patient survival associated with degree of tumour stroma immunostaining of peroxidasin (left panels) and ADAMTS16 (right panels) in lung adenocarcinoma (**a**) or lung squamous cell carcinoma (**b**) patients represented in the TMA cohort. Cores with uninterpretable immunostaining were omitted. HR, hazard ratio (with 95% confidence interval). Statistical analysis, log-rank test (*n* = 73 and 50 cores for adenocarcinoma and squamous cell carcinoma patients, respectively, for peroxidasin immunostaining and *n* = 87 and 53 cores for adenocarcinoma and squamous cell carcinoma patients, respectively, for ADAMTS16 immunostaining).

**Supplementary Table 1 Demographics of patients included in the study.**

| **Characteristic** | **Category** | **Number of patients** |
| --- | --- | --- |
| Gender | Male  Female | 14  21 |
| Age (years) | 40–49  50–59  60–69  70–79  80–89 | 1  3  10  17  3 |
| Tumour type | Adenocarcinoma  Squamous cell carcinoma  Large cell tumours  Pleomorphic | 17  12  3  2 |
| Degree of tumour differentiation | Poor  Moderate  Well  Not recorded | 13  19  1  1 |
| Tumour (T) stage | T1  T2  T3  T4 | 1  17  10  6 |
| Lymph node (N) stage | N0  N1  N2 | 24  7  3 |
| PET uptake | Low  Moderate  High  Not recorded | 0  1  32  1 |
| Smoking status | Current  Previous  Never | 9  24  1 |
| Histology non-cancerous tissue | Normal  Emphysema  Emphysema and fibrosis  Emphysema and inflammation  Emphysema and pneumonia  UIP fibrosis and emphysema  Mild emphysema and non-caseating granuloma  Inflammation  Infract and pigment laden macrophages  Pleural fibrosis  Pneumonia  Sarcoidosis  Not recorded | 7  13  1  1  3  1  1  1  1  1  1  1  1 |

**Supplementary Table 2 Recently published proteomic analyses of lung tumour tissue against which our study was compared.**

| **Study** | **Lung tissue source** | **Tissue extraction method** | **Proteolytic enzyme(s)** | **MS approach** | **Matrisome proteins identified** |
| --- | --- | --- | --- | --- | --- |
| Tenzer et al. (2016) | Twenty-one patient tumour and matched non-tumour samples from 11 patients with adenocarcinoma and 10 patients with squamous cell lung carcinoma | Whole tissue lysates concurrently used for RNA extraction | Trypsin | High-definition MS (HDMS) | 86 matrisome proteins were detected. These proteins comprised 27 glycoproteins, 1 collagen, 8 proteoglycans, 17 ECM-affiliated proteins, 24 ECM regulators and 9 secreted proteins |
| Gocheva et al. (2017) | Three KP mice, where tumour formation was stimulated using Cre-expressing adenoviruses. Tumours were graded as stage 3–4. In addition, samples were analysed from C57BL/6J mice with bleomycin-induced pulmonary fibrosis and normal lung tissues | Fractionated ECM proteins separated using a commercial subcellular compartment protein extraction kit | PNGase F, Lys-C,  trypsin | Tandem mass tag (TMT) | 100 matrisome proteins were detected initially, and 113 matrisome proteins were detected following exclusion of abundant spectra. These proteins comprised 43 glycoproteins, 30 collagens, 6 proteoglycans, 14 ECM-affiliated proteins, 14 ECM regulators and 6 secreted proteins |
| Tian et al. (2019) | Twenty patient samples with idiopathic pulmonary fibrosis and 20 control samples taken from patients undergoing surgery for pulmonary nodules/tumours | Whole tissue | Trypsin | Isobaric tags for relative and absolute quantitation (iTRAQ) | 229 matrisome proteins were detected. These proteins comprised 68 glycoproteins, 22 collagens, 14 proteoglycans, 42 ECM-affiliated proteins, 59 ECM regulators and 24 secreted proteins |

**Supplementary Table 3 Patient-derived lung cores included in the tumour microarray.**

| **Staining** | **Characteristic** | **Number of cores** | **Proportion of cores (%)** |
| --- | --- | --- | --- |
| Peroxidasin | Non-tumour (total available)  Non-tumour (interpretable)  Tumour (total available)  Tumour (interpretable)  Adenocarcinoma  Squamous cell carcinoma  Adenosquamous carcinoma  Large cell carcinoma (other)  Mixed small cell and large cell carcinoma  Pleomorphic carcinoma  Non-tumour–tumour sample pairs | 151  150  138  138^a^  73  51  5  7  1  1  119 | –  100  –  100  52.9  37.0  3.6  5.1  0.7  0.7  – |
| ADAMTS16 | Non-tumour (total available)  Non-tumour (interpretable)  Tumour (total available)  Tumour (interpretable)  Adenocarcinoma  Squamous cell carcinoma  Adenosquamous carcinoma  Large cell carcinoma (other)  Mixed small cell and large cell carcinoma  Pleomorphic carcinoma  Non-tumour–tumour sample pairs | 150  149  155  154^b^  87  53  5  7  1  1  133 | –  100  –  100  56.5  34.4  3.3  4.5  0.6  0.6  – |

^a^For tumour stroma only, 137 cores were interpretable

^b^For tumour cells only, 152 cores were interpretable
